# What Should the Dentist Do to Prevent Decay of the Teeth?

**Published:** 1896-08

**Authors:** N. S. Hoff


### What Should the Dentist Do to Prevent Decay of the Teeth?

BY N. S. HOFF, D.D.S.

The phrase " My patient " is so often encountered in conversation with professional men that the question is raised as to the actual relations of doctor and patient. There are, undoubtedly, many good reasons why his patrons and the professional man establish closer and more interdependent relationships than obtain elsewhere in the business world.

That this is a fact is evident when we recall the instances in which patients use the term " My dentist " probably more frequently than those where the dentist claims the patient.

What claims can we make to the confidence and allegiance of patients?

It will serve the purpose of our thought to briefly enumerate some of the more evident of these claims and to dwell more particularly upon such as seem to us to contain the elements which should inspire our patrons with the greatest confidence and not only merit but oblige their continued patronage.

The first and perhaps the most apparent reason is the efforts made to induce patients to make the first approach. This involves the whole question of location of one's business place and its surroundings, convenience and accessibility, attractive furnishings, ample appliances and facilities, at least an agreeable, and if possible an attractive personality combined with a thorough professional education and a happy and judicious mingling with society, business and professional people and other legitimate means for extending one's acquaintance. All of which may and should be done in an honorable and dignified way.

These things are important elements in the establishment of a dental practice and should not be neglected, but the most conclusive and commendable element is that which involves a spirit and determination to make conscientious endeavors to circumvent the ravages of disease, and in proportion as our efforts in that direction not only of repairing damages, but of warding off disease, have been successful, shall we have the moral right to expect or claim the continued patronage of those who may or would be inclined to seek our services.

This introduces the question which serves as the title of this paper, "What Should the Dentist Do to Prevent Decay of the Teeth?"

As a general answer, I should say that we should do whatever may be done to secure a faultless development and make persistent and intelligent efforts to check and ward off all destructive influences.

To secure a perfect development we will necessarily begin efforts which will look to the end, at a very early time, in the development of the teeth.

It will be a wise foresight, perhaps, to advise the mother of the importance of giving attention to her own health and well-being in order that the teeth of the offspring may be of the best character. Just how much can be done here it is difficult to say, for many practical difficulties stand in the way of offering advice of this kind. The dentist is not often, or at least always, acquainted with the circumstances which would render it possible or practicable to offer advice of this kind; or there may stand between him and his patients social or civil reserves which prevent a free discussion of such matters with them. I am, however, inclined to think that the strongest reason we can think of why more advice of this kind is not given our patients is that we are not prepared to give clear and adequate advice on this subject. While I realize that this is too true, I can not feel that there can be any great censure attached, for the reason that this subject has never been carefully worked out by any one from a dental standpoint. It is true that there has been a great deal of superficial and somewhat desultory writing in our journal about the importance of this matter and even advisory treatment of a general character.

But the field of physiological chemistry is just opening up, and until some work along this line is done in a purely scientific way, we shall still go on groping in the darkness of empiricism.

Our teachers and text-books tell us to give expectant mothers foods containing the tooth elements, phosphate and carbonate of lime. This sounds well and sometimes may be good advice, but there are recorded cases of where this advice has produced very serious complications, resulting in excessive bony development and difficult parturition, to say nothing of the impoverished nervous and muscular tissues.

The fact is we need the counsel of the expert physician in these conditions.

Dentists, from the very nature of their work and practice, can not be expected to make accurate diagnoses, nor to intelligently prescribe the proper quantity or quality of food for the great variety of physical conditions of the women of our day, but it is my opinion that we should not altogether ignore this matter, but that we should make ourselves as familiar with its general bearing as possible and advise with the family physicians of our patients and gather data that shall help to establish more definite and scientific methods of meeting this problem than any we now possess.

The dentist can render invaluable aid to the physician in this matter as he can determine results with a considerable degree of accuracy.

He would at least be able to determine the comparative value of different treatments in the results secured and be able to fairly compare or value these results.

Secondly, it seems to me that it is high time more notice was given to the eruption of the teeth in a regular order and harmonious position.

It is not often that the deciduous teeth emerge into irregular positions, but occasionally they do, and I need not tell you, gentlemen, that irregular or mal-positioned teeth constitute a menace to their integrity.

It is, perhaps, of less consequence to these teeth because, they are short-lived, but it is of considerable importance to the second or permanent set of teeth that the primary teeth should occupy their definite places and fulfill their mission or work, until such time as the permanent teeth are ready to supplant them. The second or permanent set should be brought into perfect alignment or occlusion at as early a date as possible after they emerge from the gum-tissue. The importance of this is not so fully entertained by the profession as it should be, probably for the reason that our authorities on the question of the proper time to do regulating have not been unanimous in their opinions.

One claims that as soon as it becomes evident that a tooth is intent on, or is liable, to assume a wrong position, that it should be brought by force into its proper place, taking little or no account of the completion of development of the tooth-tissues.

The other school maintains that the regulating should be postponed until the teeth which are to form the bases and keys to the arch are not only erupted but are fully developed and have assumed a definite position.

I will not weary you with the arguments advanced nor with the good illustrations to be had on both sides. In fact, the arguments are so convincing that most of us are completely "at sea" as to the best and proper time to do this work. Is this one of the questions that must remain undecided, or can we get any more decisive information by a closer and more accurate investigation? Personally, I am not a advocate of either method, but come to a decision in view of the conditions which individual cases present. I am fully convinced that changing the position of the teeth in the jaw is relatively easier of accomplishment the earlier it is undertaken, and if carefully done will be accomplished without injury.

There is this to be said in favor of early regulating: that the teeth will be better developed and consequently put into the best condition to resist the action of corrosive agents and to admit of repair when disease shall attack them, and they will be in proper position to do their part in the general nutrition of the body, and for their own good.

We can not, with these points in mind, fail to take into account the conservative value of perfectly-developed teeth and their normal position in the dental arches.

The third element in our somewhat complex review of this question will be that which takes into account the value of cleanliness, or prevention of the development of destructive agents in the mouth. A well-developed tooth-tissue will resist the attacks for a long time of the vicious influences generated or to be found in many mouths, a regular and complete denture will, to a certain extent, make it impracticable for these disease influences to generate in sufficient quantity to accomplish any considerable amount of injury. Yet, in spite of these happy influences, sooner or later, conditions will be obtained in the mouth which will call for outside, or artificial help, to overcome the evil agencies. A demand will arise for the tooth-brush, the pick, the ligature and other mechanical means for removing from the interdental spaces, fissures and surfaces of the teeth extraneous and disease-breeding deposits.

These will also need to be supplemented with various detergent and disinfectant tooth-powders, soaps and washes. It will suffice here for me to say that there is no question as to the value of the regular and intelligent use of these agents to prevent the active destruction of the teeth by the usual decay-producing agents.

Shall I take your time to detail the character of these prophylactic measures? I shall not presume to do so in detail, and yet, I would suggest that the tooth-brush should always be fitted to the mouth by the dentist rather than by the druggist or patient. This is as proper a function of the dentist as is the fitting of spectacles by the oculist. Prescribing a tooth-powder or mouth-wash should, rarely if ever, be left to the office boy or the druggist.

Every mouth does not required a radical detergent powder any more than every mouth requires a powerful disinfectant mouth-wash; each of these can do incalculable injury if used when not indicated. Irregular teeth can not be properly cleaned by a rigid, flat-faced tooth-brush, and a gritty or insoluble tooth-powder used with such a brush will waste beyond repair the soft tissues and the tooth-substance as well in all exposed or prominent places. Neither should these gritty powders or alkaline pastes or soaps be used on irritable and sensitive gum-tissues of low vitality because of impaired nutrition; rather mild stimulating and slightly astringent antiseptic washes or soluble powders should be preferred.

In every case the prevailing conditions should receive careful consideration, and should there be any tendency to degeneration nothing aggravating should be allowed, but corrective measures should be devised each for the peculiar conditions present.

It is not always easy to diagnose local conditions, as many systemic disorders manifest themselves in the mucous membranes of the mouth; but in most cases these local manifestations of very obscure disorders will at least tolerate a mild antiseptic treatment when they would rebel at the application of excessive irritants. Cleansing appliances and applications should also be made agreeable to the patient. Awkward or difficult appliances to use will not be effective, or at least will not be effectively used.

Nauseating powders or washes will not be used by children or delicate people, who generally need such things most. All mouth-washes and detergent powders, soaps or pastes should be made agreeable to the palate, so that patients will rather incline to make good and regular use of them.

Avoid bitter, hot or astringent excesses of all kinds, and if they are necessarily indicated correct the unpleasant taste or effect with sugar, to sweeten and overcome bitter elements, and bland oils or gums to emulsify the excessive irritants and so prevent excessive irritation and give them a more continuous action.

A BASIS FOR TOOTH-POWDER.

| | | | |
|---|---|---|---|
| Take of | English precipitated chalk | 10 | drachms |
| " " | finely-powdered cuttle-fish bone | 6 | " |
| " " " | " white sugar | 3 | " |
| " " " | " white castile soap | 1 | " |

Mix, color with carmine, flavor with rose or wintergreen oil, and sift through a very fine sieve. This powder will serve all practical purposes where no medical effects are desired. If an astringent effect is wanted add 15 drops of tincture of myrrh; if a stimulant and astringent effect is desired add 20 drops of tincture of myrrh and capsicum to the original formula; if an antiseptic powder is wanted add to the formula 30 drops of Black's 1, 2, 3 remedy. It may be made into a paste by mixing with it glycerine, or honey and listerine. It can be incorporated with softened castile soap and pressed into hard cakes. It may be mixed with mucilage or gum arabic and pressed into tablets. If the soap is objectionable use in place of it powdered soap-tree bark; if the sugar is objectionable use saccharin, 15 grains. If the cuttle-fish bone is too insoluble or gritty substitute carbonate of magnesium. And in various ways the powder may be modified or changed to conform to the requirements of special cases.

A BASIS FOR A TOOTH-WASH.

| | |
|---|---|
| Take of saccharin | 10 grains |
| " " bicarbonate of soda | 10 " |
| " " spirit | 10 drachms |

Warm gently to facilitate solution and combination and add 10 grains of salicylic acid. For use put a teaspoonful in a half a glass of water; this makes an alkaline, antiseptic and stimulant mouth-wash. It may be made astringent and stimulant by adding tincture of myrrh and capsicum.

It may be made more antiseptic by adding listerine. It may be more agreeable to use if one drachm of the fluid extract of soap bark be added.

In addition to the ordinary prophylactic precautions just indicated, some care is needed to overcome the incidental effects of corrosive drugs administered by the physician in the treatment of systemic conditions.

The administration of all acid preparations by the mouth should be accompanied with such precautions as may be indicated to avoid having the acid come into contact with the teeth, and in addition, a vigorous brushing of the teeth with an alkaline wash will be advisable, and the packing between the teeth of some alkaline preparation that is not readily dissolved will be very helpful; finely-prepared or precipitated chalk has been much used for this purpose; solutions of bicarbonate of soda, as a fluid wash, will meet most cases. The administration of soluble salts, such as aluminum, sodium and calcium, liberate their acids in a very active form in the mouth, and probably produce more extensive corrosive effects than free acids, because of the fact that their action is not suspected. It is a mistake to think that the salts of alkaline substances when decomposed in the mouth will themselves correct the resultant acids.

The administration of iron preparations, particularly the tincture of the chlorid, is liable to produce corrosion of the teeth by the free hydrochloric acid in the preparation. This may be counteracted by the liberal use of water and alkalies. A solution of the oxide of magnesia is much used for this purpose, as it is not readily soluble, and will keep the mouth alkaline or neutral for a considerable period.

Another important element in the preservation of the teeth is the conservation of good bodily health. Everything that will tend to make a vigorous and healthy constitution will make toward the preservation of the teeth. A strongly-fortified body means well-nourished teeth, healthy secretions from the oral tissues, good digestion and general condition of the fluids of the body which will overcome fermentation and putrefactive tendencies. This means that the various organs and tissues of the body shall be given proper exercise, and none, nor any of them, overworked.

The lungs should have plenty of pure air to keep them active. The digestive organs should have plenty of good, digestible food, of such variety as to give each organ a proportionate amount of work to do, and the food should not be excessive either for all the organs nor for any particular set. It should be eaten at suitable and regular intervals, so as to give periods of rest and work or activity. The use of stimulants, such as bitters, tonics, spices, etc., for healthy organs are not required and their use, to hurry up the action of fatigued or diseased organs, is questionable, so much so that they should never be used merely to gratify the taste, but always on the advice of a competent physician. On the same principle the use of narcotics to relieve the distress from excessive eating is equally to be condemned. All these too common practices tend to clog the body emunctories and prevent their normal functions to such an extent that the soft tissues of the body become impregnated with waste and poisonous materials, which set up disease of these tissues to such an extent as to accomplish their destruction. In the mouth we find notable examples in tumid gums, ulcerated mucous membranes, calcic deposits, and the worst of all oral diseases, pyorrhœa alveolaris. This subject is too extensive for me to more than touch upon here, and I wish only to suggest enough to make apparent the necessity for the enforcement of good hygienic principles on behalf of the dental tissues, in the way of good and pure food in proper quantities, an abundance of pure air and ample, warm and protecting clothing.

Now, if all these things are important to the minority of people who are healthy, what shall we say of the great majority who are not always in the enjoyment of perfect health, and many of them never see a well day?

Of course our line of argument will permit of no other suggestion than that they shall be restored to health, or at least, a practicable measure of it. Shall this be the work of the dentist, or must he stand by and do what he can to rescue the decaying teeth, disregarding all efforts to better the systemic conditions? We should say no; the dentist should, of course, not attempt the practice of general medicine, but should advise with the patient and his physician and see that all that could be, was done, to protect his efforts at repair.

And, having done this, and urged its importance on both his patient and the physician, he is ready to take up the last, and to some extent, the largest and most important factor in our consideration of this subject.

This is such a large subject that I shall only allude to it in concluding this paper and in a general way. At this period in dental practice no one will question the statement that repairing the ravages of disease of the teeth should always be undertaken, and pushed to the limits of one's abilities and the circumstances which will ever modify the particular forms of treatment. We are not yet able to dogmatize as to the kinds of material or manner of using this material, nor even as to the time to use it. One general rule, I think, we may adopt as a safe starting point, and that is, as soon as disease attacks the tooth it should receive some kind of treatment. The child's teeth, if they decay prematurely, must be filled, cauterized, eroded or polished, or in any way treated so as to preserve them until such time as they shall have fulfilled their full function. Incipient decay of the second or permanent teeth should have equally prompt and decided attention. The best material and most perfect adaptation is none too high a service. In fact, nothing short of a thoroughly adequate and reliable treatment will suffice. In some countries the decay of the teeth is looked upon as a universal disease which is harmless and does not jeopardize the general health, but must run its course, and when the teeth are all gone artificial ones can be had to take their places. Happily, this idea does not prevail in our country, but the dentist is expected to preserve every tooth even to the end of life. It is not too much to predict this as a general result of the wonderful advances we are making in our knowledge of treatment, and the more intelligent care which both patients and dentists are now giving, not only to the development of strong tooth-tissues, but also to their preservation by early and persistent treatment, looking to the correction of disease and the prevention of its recurring attacks. It seems to me that the important work of the dentist of to-day lies very much along the line of early and thorough operations upon the teeth. Early, to prevent the least possible loss of valuable tooth-structure; thorough, that needless repetition may be avoided, for every time a tooth is filled it is correspondingly weakened structurally and, to some extent, functionally. As a theoretical ambition or aim, which can not, however, always be made practical, I would suggest a maxim something like this: "Never temporize, but make each and every filling for life."

